# Impacts of Climate Change on Grain Production in China, Japan, and South Korea Based on an Improved Economy–Climate Model

**DOI:** 10.3390/foods14193301

**Published:** 2025-09-23

**Authors:** Haofeng Jin, Jieming Chou, Yaqi Wang, Hongze Pei, Yuan Xu

**Affiliations:** 1 Institute of Disaster Risk Science, Faculty of Geographical Science, Beijing Normal University, Beijing 100875, China; hfjin@mail.bnu.edu.cn (H.J.); 202321051172@mail.bnu.edu.cn (Y.W.); 202421051190@mail.bnu.edu.cn (H.P.); yuanxu@cma.gov.cn (Y.X.); 2 State Key Laboratory of Earth Surface Processes and Disaster Risk Reduction, Beijing Normal University, Beijing 100875, China

**Keywords:** economy–climate model, impact ratio of climate change, climate change, grain production

## Abstract

Climate change threatens grain production in East Asia. This study assesses the impacts of climate variables and climate change on rice, wheat, and maize total production using an improved economy–climate model (C-D-C model). The innovation is to model a roughly inverted U-shaped relationship between dry-wet conditions (measured by Standardized Precipitation Evapotranspiration Index, SPEI) and production. Building on this, this study introduces a new metric reflecting extent of future climate change impact, the Impact Ratio of Climate Change (IRCC), to project the impact on production under three climate scenarios (SSP1-2.6, SSP2-4.5, SSP5-8.5) for 2021–2050. Key findings include: The dry–wet conditions exhibit a significant roughly inverted U-shaped relationship with grain production in some crop areas, with optimal production levels observed near an SPEI of zero. Effective accumulated temperature positively affects wheat production in most regions, while higher effective accumulative temperatures reduce production in warm southern areas. Future climate change in 2021–2050 will likely increase rice production in northern China but decrease it in the south (IRCC > −30%). Overall impacts on wheat will be modestly negative, accounting for about 10% of future total production. Impacts in Japan and Korea will be minimal, with absolute values of IRCC not exceeding 2.5% across all scenarios.

## 1. Introduction

Agriculture is one of the industries most sensitive to climate change. Since the Industrial Revolution, climate change has had a severe negative impact on food production in most regions around the world [1,2,3]. The Food and Agriculture Organization (FAO) estimates that global grain losses in 2020 were nine times higher than in 1991 [4]. Rising average temperatures affect crop production by shortening the growing period, altering photosynthetic efficiency, and changing respiration rates [5,6,7,8]. Changes in precipitation patterns also influence water availability for crops during different growth stages [9,10,11]. Currently, most crop yield losses can be attributed to weather and climate-related disasters [4]. Grain production systems in East Asia are particularly sensitive to climate change. The region experiences unstable climatic conditions and large interannual variability [12,13]. This study focuses on China, Japan, and the Republic of Korea as they represent the core of East Asia’s food security system. China is the region’s agricultural powerhouse, whose production has been accounting for more than 70% of total production of the region since 1990s (data source: World Bank). Conversely, Japan and South Korea are highly developed nations with significant net food import needs, making their domestic production crucial for buffering against global market volatility. Therefore, assessing the impacts of the current and future climate on grain yields in China, Japan, and the Republic of Korea is crucial for providing scientific guidance for agricultural policies and food security strategies in these countries.

Statistical analysis is one of the most common methods for studying this issue. It evaluates the climatic impacts on agricultural practices and their regional variations from the perspective of real-world production activities. By selecting production (i.e., total yields) as the dependent variable and climatic factors as independent variables, regression analysis is conducted to estimate partial regression coefficients. Chou et al. introduced climate as a production factor into the Cobb–Douglas production function, constructing an economy–climate model (C-D-C model). Their simulations and validations demonstrated that this model achieves better fitting and extrapolation performances [14]. Subsequent studies likewise incorporated both socioeconomic factors and climate inputs in regression analyses to analyze the impact of average temperature, total precipitation, and so on, on grain production [15,16,17,18]. However, most existing statistical models only consider monotonic relationship between climatic variables and crop production. In reality, either excessively high or low levels of climatic factors can hinder crop growth. Thus, it is necessary to develop a statistical model able to capture this non-monotonic relationship.

This study first refines the existing economy–climate model (C-D-C model) to incorporate the non-monotonic relationship between dry-wet conditions and crop production (i.e., gross yields). The improved model is then employed to evaluate the effects of climatic factors on crop production across China, Japan, and South Korea. Additionally, the Impact Ratio of Climate Change is constructed based on the modified C-D-C model to assess the extent of future climate change impacts on production under three climate scenarios (SSP1-2.6, SSP2-4.5, and SSP5-8.5).

## 2. Materials and Methods

### 2.1. Overview of the Study Area

China, Japan, and the Republic of Korea are major grain producers in East Asia. Since the 1990s, the annual grain production of these three countries has consistently been more than 350 million tons, 10 million tons, and 4.9 million tons, respectively (data source: World Bank). However, the climatic environment in East Asia is unstable, with significant inter-annual variability. Since the mid-20th century, the number of extreme heat events, cold events and drought events in East Asia has been increasing [12]. Extreme climate events pose significant risks to grain production in these three countries.

Due to significant differences in the natural geographical environment and farming systems within China, the country is divided into different cropping areas (Table 1) [15].

### 2.2. Data

The research data include meteorological data and socio-economic data. For each provincial administrative unit or country, the average value across all grid points within the district or country was used as the sample’s value in a certain year. Historical meteorological data from 1991 to 2020 include daily downward shortwave radiation at the surface, daily maximum temperature, daily minimum temperature, and a drought index. The annual effective accumulated temperature were calculated using the CPC 0.5° × 0.5° Global Daily Gridded Temperature. The downward shortwave radiation flux reaching the surface is sourced from the ERA5 dataset, specifically the ERA5-Land monthly averaged data from 1950 to the present, with the variable “surface solar radiation downwards” (units: J/m^2^) at a resolution of 0.1° × 0.1°. By dividing the accumulated value by the accumulation period in seconds, the unit is converted to W/m^2^. The Standardized Precipitation Evapotranspiration Index (SPEI) data were obtained from the Global SPEI Dataset. The Palmer Drought Severity Index (PDSI) comes from the dataset: scPDSI for global land, with a resolution of 0.5° × 0.5°. Turning to the socio-economic data, data on fertilizer application, effective irrigated area, pesticide usage, agricultural employment, sown area, mulch film usage, and crop total production for each cropping area from 1991 to 2020 are sourced from the “China Rural Statistical Yearbook”, “Statistical Materials of 60 Years of Agriculture in New China”, and “China Statistical Yearbook”, statistical yearbooks from various provincial-level administrative regions in China, and from the Food and Agriculture Organization of the United Nations.

Future climate data include the daily maximum temperature (°C), daily minimum temperature (°C), downward shortwave radiation flux at the surface (W/m^2^), and SPEI (–) during the growing period from 2021 to 2050 under three scenarios: SSP1-2.6, SSP2-4.5, and SSP5-8.5. SSP1-2.6 represents a sustainable world with relatively low climate change challenges, in which global CO_2_ emissions are significantly reduced and reach net zero after 2050. SSP2-4.5 follows an intermediate pathway with moderate climate challenges; CO_2_ emissions remain near current levels before starting to decline in the mid-century but do not reach net zero before 2100. SSP5-8.5 assumes a development path focused on conventional economic growth, with a substantial increase in CO_2_ emissions. The NASA Earth Exchange Global Daily Downscaled Projections (NEX-GDDP-CMIP6) dataset provides bias-corrected future climate data with a resolution of 0.5° × 0.5° and high accuracy [19,20,21]. In addition, SPEI was calculated based on this dataset as part of the Global Drought Layers derived from NASA-NEX-GDDP [22]. In this study, the equally weighted average of all model simulations listed in Appendix A Table A1 is used as the projected climate data under each scenario.

### 2.3. Model Design

This study first improves the existing economy–climate model (C-D-C production function) to capture the non-monotonic relationship between dry-wet conditions and grain production, while also explaining the economic significance of each parameter. The improved C-D-C production function is employed to assess the effects of changes in climatic and socio-economic factors on grain yield and to identify the optimal SPEI for crop growth in each crop area. Second, based on the improved C-D-C production function, the study derives the Yield Impact of Climate Change (YICC) model and proposes an estimation formula for the Impact Ratio of Climate Change (IRCC). The IRCC estimates the ratio of the production change of future climate change to total future grain production, under given future levels of socio-economic agricultural input. Finally, a Peaks Over Threshold (POT) model based on the Generalized Pareto Distribution is constructed to fit the probability distribution of climate-induced yield loss. The model is used to calculate Value at Risk (VaR) and Expected Shortfall (ES), enabling an assessment of climate risk to grain yield loss under current climate conditions.

#### 2.3.1. An Improved C-D-C Production Function

The C-D-C model treated climate as inputs in the grain production process. By incorporating climatic factors into the Cobb–Douglas (C-D) production function proposed by Charles Cobb and Paul Douglas, it allowed for the estimation of the direct effects of each production factor—including socio-economic inputs and climate inputs—on grain yield [14]. The climate input term in this function represented a monotonic relationship between climatic variables and grain production. However, according to Shelford’s Law of Tolerance, when climatic factors exceed the upper tolerance limit or fall below the lower threshold of a crop, its survival and growth are hindered. This law implies that the relationship between climatic factors and grain yield is not strictly monotonic.

The relationship between dry-wet condition and grain production follows a relationship that first increases monotonically and then decreases monotonically (an approximate inverted-U-shaped relationship). Extreme drought limits the absorption of water and nutrients [23,24,25], while excessive moisture may lead to root hypoxia and disease [26,27]. Therefore, this study modified the C-D-C model to incorporate an approximate inverted-U-shaped relationship between dry-wet condition and grain production as Equation (1):

(1) $$Y=A\prod_{i=1}^{in}x_{i}^{\beta_{i}}C_{1}^{\gamma_{1}}C_{2}^{\gamma_{2}}e^{aC_{3}^{2}+bC_{3}}.$$

where *Y* represents grain total production (t). The climatic inputs in the equation include downward shortwave radiation (*C*_1_), which represents solar radiation; effective accumulated temperature (*C*_2_), which reflects thermal resources; and the drought index (*C*_3_), which represents natural water supply or environmental moisture conditions. Solar radiation not only provides energy for crop photosynthesis but also facilitates the transport of substances within the plant. Effective accumulated temperature reflects the thermal resources of a crop area and influences the rate of crop growth. The Standardized Precipitation Evapotranspiration Index (SPEI) captures the water supply–demand balance in a crop area and measures the deviation of precipitation and potential evapotranspiration from the average state, therefore indicating the degree of dry or wet. *A* value greater than 0 indicates wet conditions, while a value less than 0 indicates dry conditions. When the absolute value of the index is less than 1, the level of the dry–wet conditions is close to normal. The socio-economic input variables (*x_i_*) include the following: fertilizer application per unit area (*x*_1_, kg/ha), the proportion of effectively irrigated area plus one (*x*_2_, -, only for China’s crop areas), pesticide use per unit area (*x*_3,_ kg/ha), labor input per unit area (*x*_4_, people/ha), mulch film use per unit area (*x*_5_, kg/ha, only for China’s maize and wheat crop areas), and sown area (*x*_6_, 10^3^ ha). *A*, *β_i_*, *γ*_1_, *γ*_2_, *a*, and *b* are parameters to be estimated.

The logarithmic form of Equation (1) is as follows:

(2) $$\ln Y=\ln A+\beta_{i}\sum_{i=1}^{in}\ln x_{i}+\gamma_{1}\ln C_{1}+\gamma_{2}\ln C_{2}+aC_{3}^{2}+bC_{3}.$$

By taking the partial derivatives of *Y* with respect to *x_i_*, *C*_1_ and *C*_2_, the elasticities of yield with respect to each input can be derived. Taking the effective accumulated temperature elasticity of yield as an example:

(3) $$\frac{\partial Y}{\partial C_{2}}=A\prod_{i=1}^{in}x_{i}^{\beta_{i}}C_{1}^{\gamma_{1}}e^{aC_{3}^{2}+bC_{3}}\gamma_{2}C_{2}^{\gamma_{2}-1}.$$

The production elasticity with respect to effective accumulated temperature ($E_{C_{2}}$) is as follows:

(4) $$E_{C_{2}}=\frac{\partial Y}{\partial C_{2}}\cdot \frac{C_{2}}{Y}=\gamma_{2}.$$

This means that a 1% increase in effective accumulated temperature directly leads to a *γ*_2_% increase in production.

Taking the partial derivative of *Y* with respect to *C*_3_, Equation (5) can be obtained:

(5) $$\frac{\partial Y}{\partial C_{3}}=A\prod_{i=1}^{in}x_{i}^{\beta_{i}}C_{1}^{\gamma_{1}}C_{2}^{\gamma_{2}}e^{aC_{3}^{2}+bC_{3}}(2aC_{3}+b).$$

Thus, the semi-elasticity of production with respect to *C*_3_ ($SE_{C_{3}}$) is as follows:

(6) $$SE_{C_{3}}=\frac{\partial Y}{\partial C_{3}}\times \frac{1}{Y}=2aC_{3}+b.$$

This implies that for every one-unit increase in *C*_3_, grain production changes by (2*aC*_3_ + *b*) × 100%. When *C*_3_ = 0, the semi-elasticity of production with respect to the drought index is equal to *b*.

Let $\frac{\partial Y}{\partial C_{3}}=0$, and, therefore, $C_{3}=-\frac{b}{2a}$. Assuming *a* < 0, when $C_{3}\in (-\infty ,-\frac{b}{2a})$, then 2*aC*_3_ + *b* > 0. Based on the economic interpretation of the function, this implies that $A\prod_{i=1}^{in}x_{i}^{\beta_{i}}C_{1}^{\gamma_{1}}C_{2}^{\gamma_{2}}e^{aC_{3}^{2}+bC_{3}}>0$, and, furthermore, $\frac{\partial Y}{\partial C_{3}}>0$. This means that *Y* increases as *C*_3_ increases. Similarly, when $C_{3}\in (-\frac{b}{2a},+\infty )$ and *a* < 0, *Y* decreases as *C*_3_ increases. This indicates that the term can capture the approximate inverted-U-shaped relationship between dry-wet condition and production. When $C_{3}=-\frac{b}{2a}$, and other inputs are held constant, production reaches its maximum.

In addition, this inverse-U curve is symmetric about $C_{3}=-\frac{b}{2a}$. Let *C*_3_′ and*C*_3_*″* be two values symmetric around $C_{3}=-\frac{b}{2a}$. Substituting *C*_3_ = *C*_3_′ and *C*_3_ = *C*_3_*″* into Equation (1) gives:

(7) $$\left\{\begin{array}{l} Y\prime =A\prod_{i=1}^{in}x_{i}^{\beta_{i}}C_{1}^{\gamma_{1}}C_{2}^{\gamma_{2}}e^{aC_{3}\prime^{2}+bC_{3}\prime }\\ Y\prime \prime =A\prod_{i=1}^{in}x_{i}^{\beta_{i}}C_{1}^{\gamma_{1}}C_{2}^{\gamma_{2}}e^{aC_{3}\prime \prime^{2}+bC_{3}\prime \prime }\\ \frac{C_{3}\prime +C_{3}\prime \prime }{2}=-\frac{b}{2a} \end{array}\right..$$

Through algebraic transformation, *Y′* = *Y″* could be obtained, indicating that when the degrees of excessive dryness and excessive wetness are equal, their impacts on production are equivalent.

The values of parameters in Equation (2) were estimated using the Ordinary Least Squares (OLS) method. An F-test was conducted to check the overall significance of the model. A *t*-test was applied to the partial regression coefficients to test the significance of the linear relationship between each individual independent variable and the dependent variable.

#### 2.3.2. Yield Impact of Climate Change (YICC)

The Impact Ratio of Climate Change (IRCC), deriving from YICC, is used to estimate the extent to which future climate change will affect grain production [28]. In agricultural practice, grain production is primarily determined by farmers’ production inputs and the climatic environment. Therefore, this study ignores other influencing factors, and in Equation (1), $N(x_{1},\ldots ,x_{in})=A\prod_{i=1}^{in}x_{i}^{\beta_{i}}$, representing socio-economic and technological inputs, so that $Y=NC_{1}^{\gamma_{1}}C_{2}^{\gamma_{2}}e^{aC_{3}^{2}+bC_{3}}$.

Assuming that agricultural production inputs remain roughly stable in scale during both the previous *n* years and the subsequent *n* years, let the average conditions during the previous *n* years for production, socio-economic and technological inputs, downward shortwave radiation, effective accumulated temperature, and SPEI be denoted as *Y*_1_, *N*_1_, *C*_1,1_, *C*_2,1_ and *C*_3,1_, respectively, and let the averages during the subsequent *n* years be *Y*_2_, *N*_2_, *C*_1,2_, *C*_2,2_ and *C*_3,2_. Then:

(8) $$Y_{1}=N_{1}{C_{1,1}}^{\gamma_{1}}{C_{2,1}}^{\gamma_{2}}e^{aC_{3,1}^{2}+bC_{3,1}},$$

(9) $$Y_{2}=N_{2}{C_{1,2}}^{\gamma_{1}}{C_{2,1}}^{\gamma_{2}}e^{aC_{3,2}^{2}+bC_{3,2}}.$$

A hypothetical scenario is constructed, in which Y^*^ represents the grain production under the socio-economic level *N*_2_ during the subsequent *n* years but climate conditions in the previous *n* years:

(10) $$Y^{*}=N_{2}{C_{1,1}}^{\gamma_{2}}{C_{2,1}}^{\gamma_{2}}e^{aC_{3,1}^{2}+bC_{3,1}}.$$

Subtracting Equation (10) from Equation (9) gives the amount of grain production in the subsequent *n* years that is attributable solely to climate change, referred to as the Yield Impact of Climate Change (YICC), denoted as Δ*Y*:

(11) $$\begin{array}{ll} \Lambda Y & =Y_{2}-Y^{*}=N_{2}({C_{1,2}}^{\gamma_{1}}{C_{2,2}}^{\gamma_{2}}e^{aC_{3,2}^{2}+bC_{3,2}}-{C_{1,1}}^{\gamma_{1}}{C_{2,1}}^{\gamma_{2}}e^{aC_{3,1}^{2}+bC_{3,1}})\\ & =Y_{2}\times \frac{{C_{1,2}}^{\gamma_{1}}{C_{2,2}}^{\gamma_{2}}e^{aC_{3,2}^{2}+bC_{3,2}}-{C_{1,1}}^{\gamma_{1}}{C_{2,1}}^{\gamma_{2}}e^{aC_{3,1}^{2}+bC_{3,1}}}{{C_{1,2}}^{\gamma_{1}}{C_{2,2}}^{\gamma_{2}}e^{aC_{3,2}^{2}+bC_{3,2}}}. \end{array}.$$

By performing algebraic transformation on Equation (11), Equation (12) is derived, which defines the Impact Ratio of Climate Change (IRCC):

(12) $$IRCC=\frac{\Delta Y}{Y_{2}}=\frac{C_{1,2}^{\gamma_{1}}C_{2,2}^{\gamma_{2}}e^{aC_{3,2}^{2}+bC_{3,2}}-C_{1,1}^{\gamma_{1}}C_{2,1}^{\gamma_{2}}e^{aC_{3,1}^{2}+bC_{3,1}}}{C_{1,2}^{\gamma_{1}}C_{2,2}^{\gamma_{2}}e^{aC_{3,2}^{2}+bC_{3,2}}}.$$

The IRCC represents the ratio of production directly affected by climate change to actual production in the future. It serves as an efficiency indicator to measure the impact of climate change on economic output. Using this indicator, the study analyzes the sensitivity of future grain production (2021–2050) under three scenarios (SSP1-2.6, SSP2-4.5, and SSP5-8.5) to climate change relative to the mean state of climate during the period from 1991 to 2020. It is worth noting that this indicator cleverly bypasses the challenge of forecasting future socio-economic agricultural inputs and only requires climate data.

#### 2.3.3. Model Validation Approaches

(1) Validation approach of the improved C-D-C production function

This section is to test fitness and extrapolation performance of the model (2). In order to test the fitting performance, the Residual Sum of Squares (RSS), Root Mean Square Error (RMSE), and Adjusted Coefficient of Determination (Adjusted R^2^) are calculated based on the sample data from 1991 to 2020 for each production region.

In addition to evaluating the model’s fitting performance, this study also validated its extrapolation performance to ensure that the model generalizes well to unseen data and does not suffer from overfitting. For the crop areas in China, which have relatively large sample sizes, the data were randomly split into a training set (70%) and a test set (30%). The model was fitted and parameters were estimated using the training set, and its performance was validated on the test set by calculating the Taylor Skill Score (TSS). Three experiments were conducted for each Chinese crop area. For the crop areas in Japan and Korea, which have smaller sample sizes, leave-one-out cross-validation was adopted due to limited samples. One sample was selected as the test sample, while the remaining samples were used to fit the model. The model was then validated on the test sample by calculating its relative error. This procedure was repeated as many times as the number of samples, with each sample used once as the test set. The mean of all relative errors—the Mean Relative Error (MRE)—was used to evaluate the model’s extrapolation performance in Japan and Korea. The standard deviation of the relative errors (SD_RE_) was also calculated to assess the stability of this performance indicator.

Taylor Skill Scores (TSSs) are commonly adopted in atmospheric sciences to compare model performance [29]. Compared to traditional single-indicator evaluation methods, the TSS provides a more comprehensive assessment [30]. It simultaneously calculated the difference in standard deviation between the model-simulated and observed data from the perspective of amplitude, which accounts for the consistency between simulation and observation, and the correlation coefficient to reflect the ability to capture patterns [29].

(13) $$TSS=\frac{4(1+R)}{{\left(\frac{\sigma_{f}}{\sigma_{r}}+\frac{\sigma_{r}}{\sigma_{f}}\right)}^{2}{\left(1+R_{0}\right)}^{4}}.$$

The range of the Taylor Skill Score (*TSS*) is [0, 1], with values closer to 1 indicating stronger extrapolation capability of the model. *R* represents the correlation coefficient between the simulated values and the observed (i.e., actual or reference) values; *σ_f_* denotes the standard deviation of the simulated data, and *σᵣ* denotes the standard deviation of the observed data. *R*_0_ represents the correlation coefficient under the ideal condition between simulation and observation, which was set to 1 in this study. The calculation method of the correlation coefficient and its relationship with the Centered Root-Mean-Square Difference (*CRMSD*) are shown in Equations (14) and (15).

(14) $$R=\frac{\frac{1}{N}\sum_{n=1}^{N}\left(f_{n}-\bar{f})(r_{n}-\bar{r}\right)}{\sigma_{f}\sigma_{r}}.$$

(15) $$\left\{\begin{array}{l} CRMSD=\sqrt{\frac{1}{N}\sum_{n=1}^{N}{\left[(f_{n}-\bar{f})-(r_{n}-\bar{r})\right]}^{2}}\\ CRMSD^{2}=\sigma_{f}^{2}+\sigma_{r}^{2}-2\sigma_{f}\sigma_{r}R \end{array}\right..$$

where *N* represents the size of test set, *f_n_* represents the simulated value of the *n*th sample while *r_n_* represents the observed (i.e., statistical) value of the *n*th sample.

Leave-one-out method and the Mean Relative Error (*MRE*) averaged over *N* experiments are adopted to assess extrapolation performance of the model for Japan and South Korea, as shown in Equation (16).

(16) $$MRE=\frac{1}{N}\sum_{i=1}^{N}\left|\frac{r_{i}-f_{i}}{r_{i}}\right|.$$

where *r_i_* represents the observed value in the *i*^th^ experiment for testing, while *f_i_* represents the simulated value in the *i*^th^ experiment for testing. A total of *N* experiments were conducted.

(2) Validation approach of YICC

This section is to introduce detailed approach by which the accuracy of the equations for Yield Impact of Climate Change (YICC) and Impact Ratio of Climate Change (IRCC) is validated, given in Equations (11) and (12) based on real-world data. Due to the limited sample sizes in the crop areas of Japan and South Korea, data from various crop areas in China were used for the validation.

The period from 1991 to 2020 was selected and divided into two stages (the first *n* years and the last *n* years). The accuracy of the calculation methods for YICC and IRCC was validated by comparing the actual values of YICC and IRCC with the estimated values from the first line of Equations (11) and (12). YICC represents the change in grain yield caused by climate change under a certain level of socioeconomic agricultural inputs. IRCC refers to the proportion of the climate-induced yield change in the actual future grain yield. According to this definition, the YICC for the future *n* years (relative to the climatic conditions of the historical *n* years) can be expressed as Δ*Y*:

(17) $$\begin{array}{l} \Delta Y=Y(N_{2},C_{1,2},C_{2,2},C_{3,2})-Y(N_{2},C_{1,1},C_{2,1},C_{3,1})\\ \;\;\;\;\;=Y(N_{2},C_{1,1}+\Delta C_{1},C_{2,1}+\Delta C_{2},C_{3,1}+\Delta C_{3})-Y(N_{2},C_{1,1},C_{2,1},C_{3,1})\\ \;\;\;\;\;=Y(N_{2},C_{1,1},C_{2,1},C_{3,1})+{\left.\frac{\partial Y}{\partial C_{1}}\right|}_{C_{1}=C_{1,1}}\Delta C_{1}+{\left.\frac{\partial Y}{\partial C_{2}}\right|}_{C_{2}=C_{2,1}}\Delta C_{2}+{\left.\frac{\partial Y}{\partial C_{3}}\right|}_{C_{3}=C_{3,1}}\Delta C_{3}\\ \;\;\;\;\;+o(\left\|\left(\Delta C_{1},\Delta C_{2},\Delta C_{3}\right)\right\|)-Y(N_{2},C_{1,1},C_{2,1},C_{3,1})\\ \;\;\;\;\;\approx \sum_{i=1}^{3}{\left.\frac{\partial Y}{\partial C_{i}}\right|}_{C_{i}=C_{i,1}}\Delta C_{i}(\mathrm{When}\sqrt{\Delta C_{1}^{2}+\Delta C_{2}^{2}+\Delta C_{3}^{2}}\to 0). \end{array}$$

The function *Y*(*N*_2_,*C*_1,2_,*C*_2,2_,*C*_3,2_) can be approximated using a first-order Taylor expansion. When $\sqrt{\Delta C_{1}^{2}+\Delta C_{2}^{2}+\Delta C_{3}^{2}}$→ 0, higher-order infinitesimal terms can be neglected, which allows an approximate calculation of Δ*Y*. In fact, compared to the variation in socio-economic inputs, changes in the climatic state are relatively small and can be regarded as infinitesimal increments approaching zero. Therefore, this study treats the last line of Equation (17) as the calculation method for the true value of the Yield Impact of Climate Change (YICC).

The periods 1991–2003 and 2008–2020 were designated as the earlier and later stages of agricultural development, respectively. These two stages differ significantly in terms of agricultural taxation policy. Since 2006, China has fully abolished the agricultural tax, leading to a dramatic shift in agricultural production inputs. Compared to 1999—the year before the pilot reform of rural taxation began—Chinese farmers experienced an estimated total reduction in tax burden of over CNY 100 billion per year after 2006, with an average reduction of approximately CNY 120 per person (source: Chinese Xinhua News Agency). This increase in farmers’ income encouraged greater investment in agricultural production. Therefore, this study assumes that the periods before and after the abolition of the agricultural tax are characterized by significant differences in socio-economic inputs into agriculture, marking two distinct development stages. Given that policy implementation and farmers’ responses are gradual processes, this study considered the year 2008 as the beginning of the later stage. Meanwhile, as the reform of agricultural taxation entered a deepening phase in 2004, the period 1991–2003 is defined as the earlier stage.

Δ*C_i_* represents the difference in the average value of climate variable *i* between two periods, namley 2008–2020 and 1991–2003, and is available. The partial derivative of grain yield *Y* with respect to each climate variable *C_i_* at the point *C_i_*_,1_ (*i* = 1, 2, 3) ${\left.\frac{\partial Y}{\partial C_{i}}\right|}_{\begin{array}{l} N_{2}\\ C_{1}=C_{1,1}\\ C_{2}=C_{2,1}\\ C_{3}=C_{3,1} \end{array}}$(*i* = 1, 2, 3) can also be calculated according to the following approach. The main idea is to interpolate the values of *Y* at four points—*Y*_inter,0_, *Y*_inter,1_, *Y*_inter,2_, and *Y*_inter,3_—based on all provincial samples within each crop area from 1991 to 2020. Here, $\bar{N_{2}}$ represents the average level of socio-economic inputs for a given province during 2008–2020, while $\bar{C_{1,1}}$, $\bar{C_{2,1}}$ and $\bar{C_{3,1}}$ represent the average values of the three climate variables for that province during 1991–2003. The partial derivatives can then be calculated based on these interpolated values.

(18) $${\left.\frac{\partial Y}{\partial C_{i}}\right|}_{\begin{array}{l} N_{2}\\ C_{1}=C_{1,1}\\ C_{2}=C_{2,1}\\ C_{3}=C_{3,1} \end{array}}=\frac{Y_{\mathrm{int}er,i}-Y_{\mathrm{int}er,0}}{0.01}$$

Radial Basis Function (RBF) was employed for interpolation. RBF interpolation is a high-accuracy method suitable for scattered data in high-dimensional spaces. Its core idea is to approximate the target function using a linear combination of a set of radially symmetric basis functions. Let $x=(x_{1},x_{2},x_{3}\ldots ,x_{6},C_{1},C_{2},C_{3})$ denote a point in an 8- or 9-dimensional space (8-dimensional for rice while 9-dimensional for wheat and maize). Suppose there are *n* observation points within a given crop area, each with a corresponding yield value *y_i_*. The mathematical formulation of the RBF interpolation can be expressed as follows:

(19) $$\overset{\wedge }{f}(x)=\sum_{i=1}^{n}\omega_{i}\varphi (\left\|x-x_{i}\right\|)$$

Here, *φ* (·) denotes the Radial Basis Function. Given the high dimensionality of the interpolation space in this study, the multiquadric (MQ) function was chosen, which is defined as $\varphi (r)=\sqrt{1+\frac{r^{2}}{\sigma^{2}}}$, where the shape parameter *σ* controls the steepness of the function. The default value from function file [31] was used. $\left\|\;\cdot \;\right\|$ represents the Euclidean distance between two points, and *ω_i_* are the weights to be determined by solving a system of linear equations.

The actual value of the IRCC can be calculated as the ratio of the actual YICC to the average crop production of the province during 2008–2020.

## 3. Results

### 3.1. Validation Results

(1) Validation results of the improved C-D-C production function

**Figure 1:**
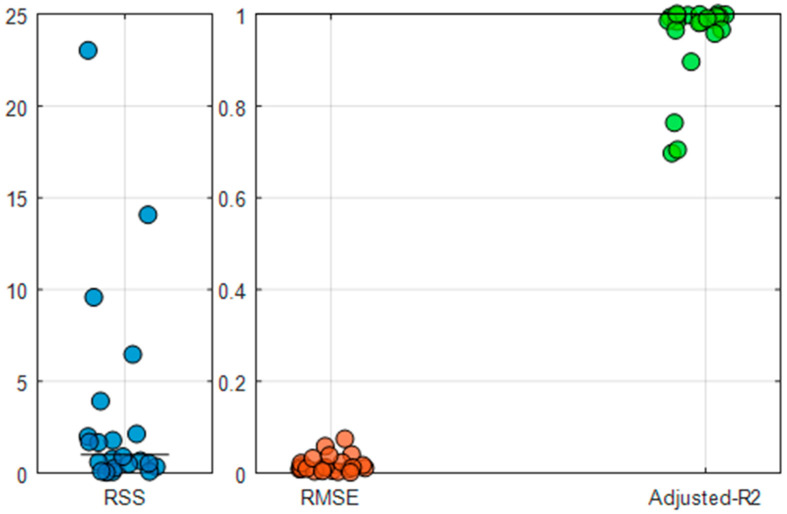
Evaluation of fitting performance of the improved C-D-C production function. Each dot represents the result of Residual Sum of Squares (RSS), Root-Mean-Square Error (RMSE) or Adjusted Coefficient of Determination (Adjusted-R^2^) in a crop area.

The results of fitting performance are shown in Figure 1. The improved model performs well across all regions. The RMSE in all regions is below 0.075, and the Adjusted R^2^ is above 0.7. In most regions, the RSS values are relatively low.

The results of extrapolation performance are shown in Table 2, and the improved C-D-C model demonstrates strong extrapolation ability. In all China’s crop areas, the TSS values from the three experiments are all greater than 0.9. For the rice and maize areas in Japan and Korea, the MREs are all below 0.1, while those in wheat areas are around 0.1. The standard deviations of the relative errors are small across all crop zones, indicating stable performance across experiments.

(2) Validation results of the improved C-D-C production function

**Figure 2:**
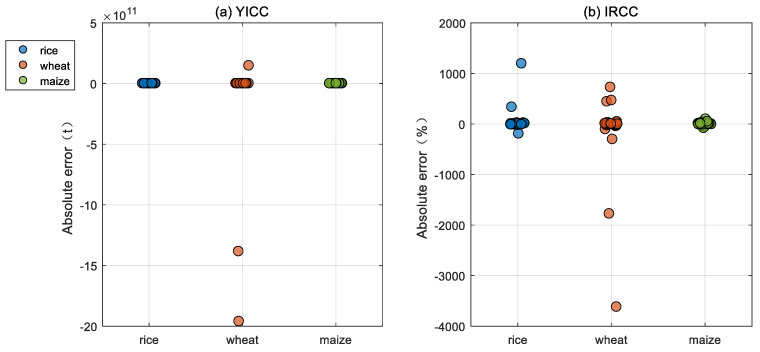
Absolute errors between actual and estimated values of Yield Impact of Climate Change (YICC) and Impact Ratio of Climate Change (IRCC). Each plot represents the absolute error of (**a**) YICC or (**b**) IRCC for a crop area.

As shown in Figure 2, the estimated YICC and IRCC exhibit small absolute errors compared to the actual values in most provinces of China, indicating that the use of Equations (11) and (12) to calculate YICC and IRCC is reliable. It is worth noting that this method tends to underestimate YICC and IRCC in most provinces. Therefore, the actual IRCC values in China may be larger than the estimated values derived from Equation (12). However, some provinces show substantial estimation errors. For instance, the estimated YICC for wheat in Qinghai and Xinjiang, as well as the IRCC for wheat in Fujian and Guangdong, are relatively inaccurate, with absolute errors of −1.4 × 10^12^ t, −2 × 10^12^ t, −3621%, and −1777%, respectively.

### 3.2. Impacts of Climate Factors on Grain Production

The improved C-D-C production function was estimated using ordinary least squares (OLS) based on samples from 1991 to 2020, and hypothesis tests were conducted to analyze the impacts of climate and socio-economic factors on grain yields in China, Japan, and South Korea.

**Figure 3:**
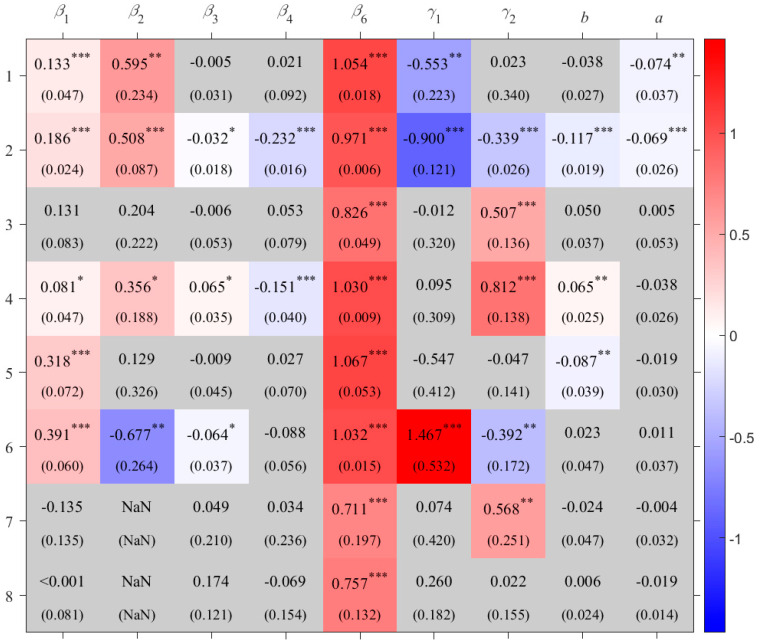
Coefficients for rice crop areas with standard error in the parenthesis. “***” indicates *p <* 0.01, “**” indicates *p* < 0.05, and “*” indicates *p* < 0.1 based on the *t*-test; grey shading indicates that the coefficient is not statistically significant at the 0.1 level, and thus the null hypothesis (that the coefficient equals zero) cannot be rejected. The numbers on the left indicate crop area codes, as listed in Table 1. The values inside the cells are the estimated coefficients; “NaN” indicates that the corresponding term is not included in the model.

For rice, the impacts of effective accumulated temperature and downward shortwave radiation on production show clear regional heterogeneity (Figure 3). In the single-cropping rice area in dry area of Northwest China (R_DNWC), a 1% increase in downward shortwave radiation is associated with a 1.47% increase in yield. However, in the double-cropping rice area in South China (R_SC) and the single- and double-cropping rice area in Central China (R_CC), increases in downward shortwave radiation have negative effects on production. This may be because, compared to the northwest, these regions have higher average temperatures, and additional radiation could trigger or intensify heat stress, thereby reducing rice yields [32,33]. In the single- and double-cropping rice area on the Southwest Plateau of China (R_SWPC), the single-cropping rice area in North China (R_NC), and the Japan rice area (R_J), increased effective accumulated temperature contributes positively to production. This effect is most pronounced in R_NC, where a 1% rise in effective accumulated temperature leads to a 0.81% yield increase. In contrast, increased effective accumulated temperature results in yield losses in R_CC and R_DNWC.

**Figure 4:**
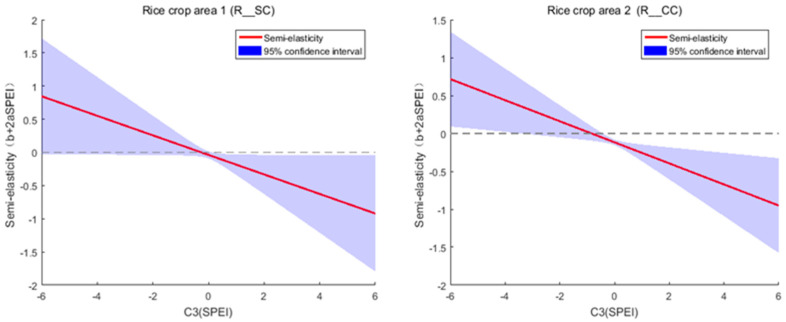
Curves of semi-elasticity of rice production with respect to Standardized Precipitation Evapotranspiration Index (SPEI) for double-cropping rice area in South China and single- and double-cropping rice area in Central China.

In the double-cropping rice area in South China (R_SC) and the single- and double-cropping rice area in Central China (R_CC), the relationship between dry–wet condition and rice production is non-monotonic. The coefficients of the quadratic terms (*a*) for these two crop areas are significantly negative, indicating that under excessively dry conditions, an increase in the Standardized Precipitation Evapotranspiration Index (SPEI) can raise rice production, whereas under excessively wet conditions, a decrease in SPEI can reduce production (Figure 4). Moreover, $1<-\frac{b}{2a}<0$ suggests that, holding other inputs constant, a slightly dry environment leads to the highest rice production in these two crop areas, with optimal SPEI values of –0.256 and –0.844, respectively.

**Figure 5:**
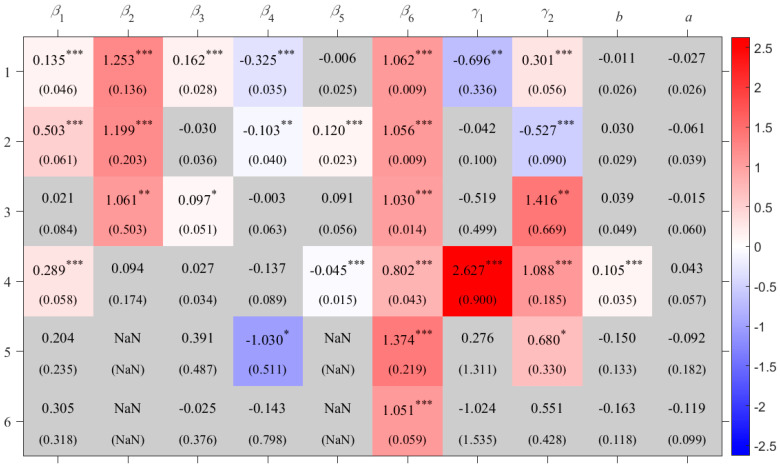
Coefficients for wheat crop areas. The caption is same as Figure 3.

For wheat, as shown in Figure 5, except for the winter wheat (autumn sowing) area in Southern China (WW_SC), effective accumulated temperature has a positive effect on total production in most crop areas. The strongest impact is observed in the spring wheat (spring sowing) area of China (SW_C), where a 1% increase in effective accumulated temperature during the growing season leads to a 1.42% increase in total production. This is followed by the winter and spring sowing wheat areas of China (WSW_C), with a 1% increase in effective accumulated temperature associated with a 1.01% increase in total production. In general, the effect of effective accumulated temperature on spring wheat production is greater than that on winter wheat.

However, the impact of incoming shortwave radiation exhibits clear regional heterogeneity. It has a substantial positive effect in the winter and spring sowing wheat areas of China (WSW_C), where a 1% increase in total shortwave radiation reaching the ground per unit area during the growing season results in a 2.63% increase in production. In contrast, it has a negative effect in the winter wheat (autumn sowing) area in Northern China (WW_NC).

Based on the sample data used in this study, no definitive conclusion can be drawn regarding the effects of dry–wet conditions on wheat production. This may be related to wheat’s strong drought tolerance.

**Figure 6:**
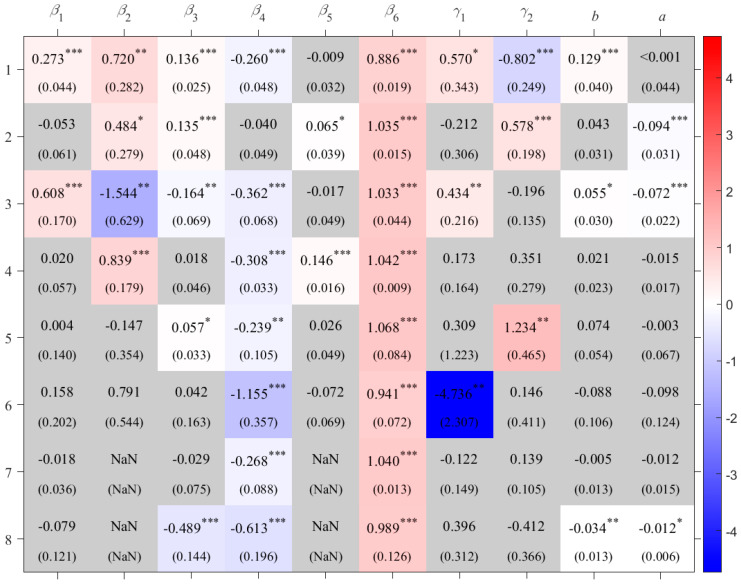
Coefficients for maize crop areas. The caption is same as Figure 3.

For maize, as shown in Figure 6, the effects of effective accumulated temperature and incoming shortwave radiation on total production also exhibit regional heterogeneity. Solar radiation has a positive impact on maize production in the maize area in the Southwest China Mountains (M_SWCM) and the spring maize area in Northern China (M_NC), but a negative impact in the Qinghai–Tibetan Plateau maize area (M_QTPC). Effective accumulated temperature has a significant negative effect on production in M_NC, where a 1% increase in effective accumulated temperature during the growing season reduces total production by 0.80%. In contrast, its impact is positive in the summer maize area on the Huang-Huai-Hai Plain (M_HHHP) and the irrigated maize area in Northwest China (M_NWC). In particular, the effect is strongest in M_NWC, where a 1% increase in effective accumulated temperature leads to a 1.23% increase in total production.

**Figure 7:**
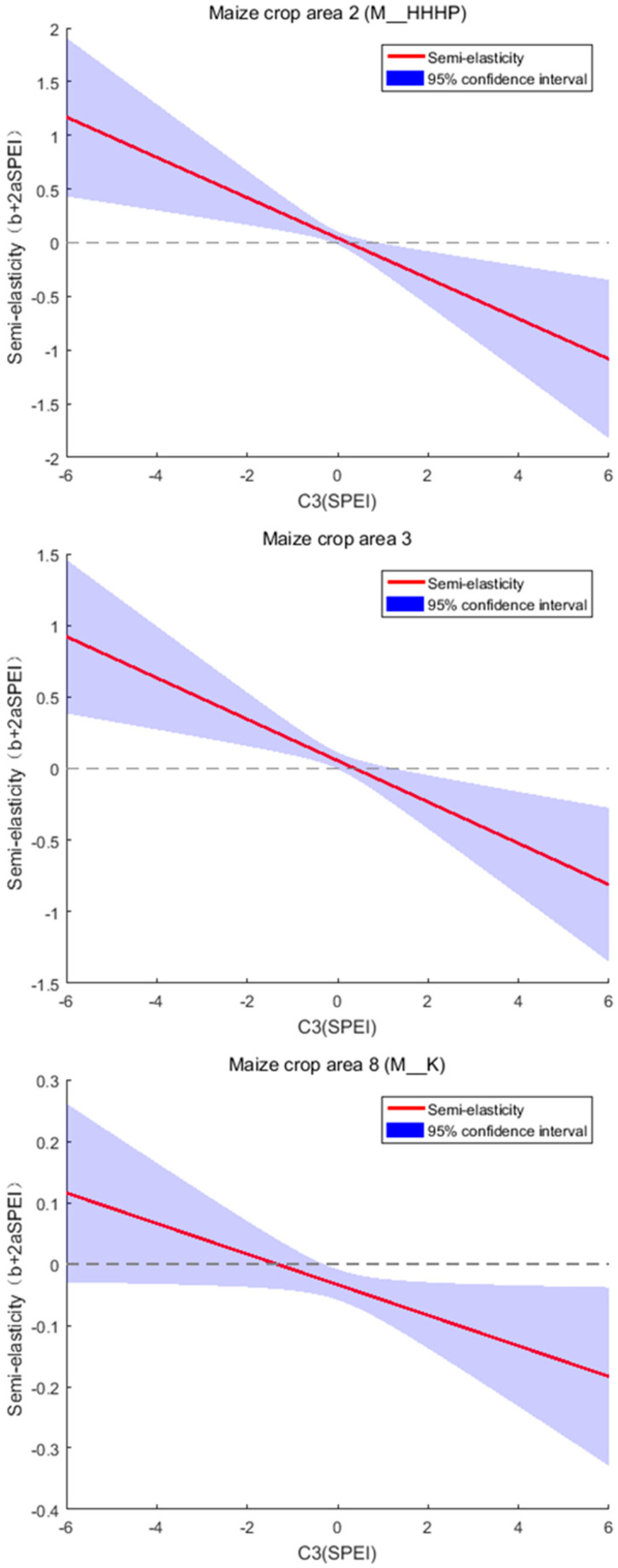
Curves of semi-elasticity of maize production with respect to SPEI for summer maize area in the Huang-Huai-Hai Plain, the maize area in the Southwest China Mountains, and the Korea maize area.

Maize production in the summer maize area on the Huang-Huai-Hai Plain (M_HHHP), the Southwest China Mountains (M_SWCM), and the Korea maize area (M_K) is significantly affected by dry–wet condition. As shown in Figure 7, in these three crop areas, maize production increases with rising SPEI when SPEI is below 0.230, 0.382, and –1.353, respectively; conversely, production increases with decreasing SPEI when SPEI is above the three levels, respectively. When facing severe drought or wet conditions, the impact on maize production is greater in M_HHHP and M_SWCM than in M_K.

### 3.3. Impact Ratio of Climate Change (IRCC) Under Future Climate Scenarios

As demonstrated in the previous section, the calculation of the Impact Ratio of Climate Change (IRCC) using Equation (12) is reliable. Therefore, based on data of historical climate (1991–2020) and multi-model ensemble mean of future projections (2021–2050), this section calculates the IRCC of rice, wheat, and maize production for each provincial-level administrative division in China, and national level in Japan and the Republic of Korea, under three different SSP scenarios: SSP1-2.6, SSP2-4.5, and SSP5-8.5.

It is worth noting that some of the estimated partial regression coefficients of climatic variables did not pass the significance test. These insignificant coefficients were set to zero in the IRCC calculation, rather than using their estimated values. This is because a *p*-value above the significance level indicates that we fail to reject the null hypothesis (i.e., the coefficient equals zero). However, this does not imply that the study considers these climatic variables have no effect on production in the corresponding crop areas; rather, it suggests that their relationship with production has not been identified within the current sample data, and thus the coefficients are treated as zero in the IRCC computation.

**Figure 8:**
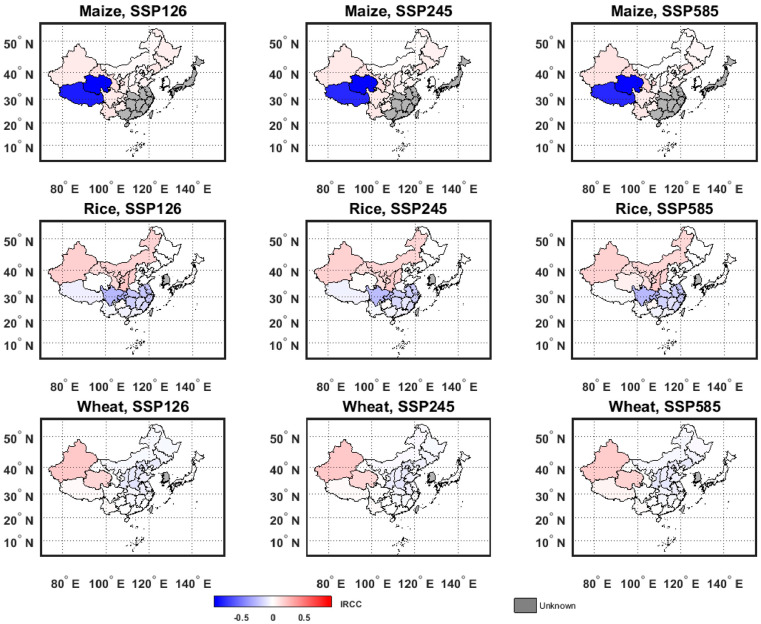
IRCC under three climate scenarios. Red indicates a positive IRCC value, with darker shades representing larger values; blue indicates a negative IRCC value, with darker shades representing smaller values; gray indicates that the coefficients of all climate factors in the region are not significant, making IRCC calculation impossible in this research.

As shown in Figure 8, during 2021–2050, climate change is projected to have a positive impact on maize production in most crop areas. Notably, the spring maize area in northern China (M_NC), the Southwest China Mountains (M_SWCM), and the irrigated maize area in Northwest China (M_NWC) are expected to experience relatively strong positive impacts, with IRCC values around 10% in most provinces within these regions. In contrast, the summer maize area on the Huang-Huai-Hai Plain (M_HHHP) shows smaller impacts, with an IRCC of around 3%. However, climate change is projected to have a substantial negative impact on maize production in the Qinghai–Tibetan Plateau area (M_QTPC), where reductions in production due to climate change will account for more than 75% of total future output. Under SSP1-2.6, the negative impact is greatest, with IRCCs in Tibet and Qinghai reaching −82% and −95%, respectively. While maize production in the Korea maize area (M_K) is also negatively affected by climate change, the extent of this impact is small, with IRCCs under all three scenarios no lower than −1%.

Climate change is projected to have a clear north–south disparity in its impact on rice production in China—mostly positive in the northern crop areas and mostly negative in the southern ones. During 2021–2050, the single- and double-cropping rice area in Central China (R_CC) is projected to face notable negative impacts, with IRCC values ranging from −10% to −20% in most provinces. In Chongqing and Sichuan, IRCCs are below −25% across all three SSP scenarios. The negative impact is smallest under SSP2-4.5, followed by SSP5-8.5, and greatest under SSP1-2.6. Conversely, climate change is projected to have a large positive impact on rice production in the early-maturing single-cropping rice area in Northeast China (R_NEC), where all provincial-level divisions show IRCCs greater than 10% across the three scenarios. The most positive impact in this region occurs under SSP1-2.6. Meanwhile, the effects of climate change on rice production in other rice areas in China and in Japan (R_J) are relatively minor.

From 2021 to 2050, the impact of climate change on wheat production is generally negative. However, the negative effects account for only a small share of total future production, with IRCC values in all areas and scenarios above −10%, except for Beijing and Henan under the SSP1-2.6 scenario. On the other hand, climate change is projected to have a positive impact on wheat production in the winter and spring sowing wheat areas of China (WSW_C), especially in Qinghai and Xinjiang, where IRCCs exceed 15% and 20%, respectively. In Japan, wheat production is only slightly affected by climate change, and the direction of impact varies across the different scenarios.

## 4. Sensitivity Test

Although considerable effort was made to include a comprehensive set of input variables that influence grain yield as explanatory variables (i.e., independent variables), estimation bias may still arise due to various reasons. Therefore, this study conducts two types of robustness checks to assess whether the newly estimated coefficients differ significantly from those presented in Figure 3, Figure 5, and Figure 6.

### 4.1. Supplementary Control for Omitted Variables

Cropping system and agricultural management are shaped by long-term local climate conditions and influence grain production. This may cause endogeneity problems, leading to the estimation bias of coefficients. Although the classification of agricultural areas in China was designed to minimize intra-zone differences in cropping systems and climate types across provincial-level administrative regions (hereinafter referred to as “provinces”), it is undeniable that differences in economic development, administrative capacity, and social conditions still lead to variations in farming practices. These differences in cropping systems and agricultural management across provinces are time-invariant provincial-level factors. To avoid estimation bias caused by the omitted variables, this study introduces a series of provincial dummy variables as control variables to account for unobserved provincial characteristics—thus constructing a provincial fixed-effects model. The within estimator is employed to estimate the partial regression coefficients of each input factor.

(20) $$\ln Y_{pt}=\ln A+\beta_{i}\sum_{i=1}^{in}x_{i,pt}+\gamma_{1}\ln C_{1,pt}+\gamma_{2}\ln C_{2,pt}+a{C_{3,pt}}^{2}+bC_{3,pt}+\mu_{p}+\varepsilon_{pt}$$

where subscript *p* denotes province *p* within the same agricultural area; *t* denotes year *t* (*t* = 1, 2, …, 30); *Y_pt_* represents the grain production of province *p* in year *t*; *μ_p_* denotes the provincial fixed effects; and *ε_pt_* is the error term.

The estimation results are shown in Appendix C Table A3. Compared with the results in Figure 3, Figure 5 and Figure 6, there are no substantial differences, indicating that the original model and its results are relatively robust.

### 4.2. Replacing the Explanatory Variable

To assess regional dry–wet conditions and monitor drought and its variation, scientists have developed various drought indices based on their specific research needs [34]. The Palmer Drought Severity Index (PDSI) is a widely used indicator in fields such as agriculture and water resources to evaluate dry–wet conditions. It reflects the water supply-demand balance in a region by calculating the difference between actual precipitation and “climatically appropriate precipitation” (i.e., water demand from soil). A positive PDSI indicates wet conditions, while a negative value indicates drought [35].

In this robustness check, the PDSI is used to replace the Standardized Precipitation Evapotranspiration Index (SPEI) as the variable representing environmental moisture conditions (*C*_3_). Using the same sample and model specification, partial regression coefficients are re-estimated. The results, presented in Appendix C Table A4, show no substantial difference from those in Figure 3, Figure 5 and Figure 6, indicating that the original model and results are relatively robust.

## 5. Discussion

The relationship between the dry–wet condition and total crop production is not monotonic. Agronomic experiments conducted in various regions have shown that alternating soil moisture levels are conducive to increasing rice yield per unit area. Such soil water conditions enhance crop production by promoting root activity and facilitating the redistribution of photosynthetic products in rice plants [36,37,38]. These findings are consistent with the conclusion of this study. Based on data from 1991 to 2020, we estimated the optimal SPEI values for crop production in R_SC, R_CC, M_HHHP, M_SWCM, and M_K. Accordingly, farmers may adopt alternating wetting and drying irrigation strategies in agricultural practice. This approach not only improves water use efficiency and reduces irrigation water consumption but also helps maintain or increase crop production [39,40]. However, both “dry” and “wet” conditions must be moderate, falling inside the optimal range. Otherwise, crop development is hindered, leading to reduced production.

The impact of effective accumulated temperature on rice and maize production exhibits spatial heterogeneity. From the perspective of average temperature, warming is generally beneficial in cold regions but detrimental in already warm regions [18]. In warmer areas, thermal conditions are already sufficient for crops to complete their life cycles, so further increases in temperature may shorten the growing period and consequently reduce total production [41]. Moreover, excessively high temperatures can inhibit vernalization, making it difficult for crops to enter the reproductive growth stage. This aligns with our findings for winter wheat in southern China and rice in Central China. In contrast, crop growth in colder cultivation areas is often constrained by insufficient thermal conditions. Warming helps meet the thermal requirements necessary for normal crop development. This is consistent with our findings for winter wheat in northern China, rice in northern China and the Qinghai–Tibetan Plateau, and maize in Northwest China and the Huang-Huai-Hai Plain.

Future climate change will have regionally differentiated effects on rice production in China, while its impact on Japan is generally positive, which is consistent with previous studies [42]. Rice production is projected to increase in Northwest China, possibly because the warming and wetting trend in this region will create favorable conditions for rice growth, whereas production is projected to decrease in southeastern China [43]. The positive effects of rising average temperatures in the future may be partly explained by elevated CO_2_ concentrations, as the CO_2_ fertilization effect promotes crop growth. Under future climate scenarios, the reproductive growth period of winter wheat is expected to be extended, while that of summer maize will be shortened. Regarding the spatial patterns of maize and wheat, our findings are consistent with previous studies [43]. However, Xiao et al. argued that under the RCP4.5 and RCP8.5 scenarios, climate change will have a negative impact on maize production but a positive impact on wheat production in the North China Plain [44,45]. This differs from the findings of our study, which may be due to differences in the reference baseline. That study compared future grain production directly with present-day production without accounting for changes in socioeconomic development, whereas our study compares future actual grain production with a hypothetical scenario in which socioeconomic factors evolve while climate conditions remain unchanged.

Our modeling framework, while effective in capturing the influences of key climate variables and factor inputs, has several important limitations that could lead to biased estimates of results. First, the models do not explicitly account for the nonlinear impacts of extreme climate events. This omission likely leads to an underestimation of future production losses, as our smoothed climatic relationships cannot capture the catastrophic crop failure associated with these extremes. Second, the C-D-C model captures output growth driven primarily by increases in input quantities (e.g., land, labor, capital, and climate) but does not incorporate growth in total factor productivity (TFP) whose growth is intensive growth driven by factors such as application of new varieties and better institutional management. By omitting this trend, our YICC model derived from the C-D-C model may overestimate the negative impacts of future climate change.

The patterns identified in East Asia suggest the models have high potential for application to other major crop-producing regions globally. Dry–wet conditions play an important role in crop yield in other crop regions of the world. For instance, in the US Midwest, maize yield is highly sensitive to soil moisture, with both drought and excess moisture causing significant reductions during the critical mid-season period [46,47]. Similarly, in Spain, soil moisture availability has been shown to outweigh the influence of other climatic factors on cereal yields during spring growth stages [48]. These findings underscore the value of applying our economy–climate model to other regions to quantify optimal dry–wet levels and identify the impacts of hydrological variability.

Furthermore, future yield projections, particularly under SSP5-8.5, remain uncertain, as evidenced by divergent projections for major wheat producers like Germany, Russia, and the USA [1]. Since most research is agronomy-based, it is necessary to apply the economy–climate model to evaluate future climate impacts and provide a complementary perspective, helping to constrain these projections and contribute to a more robust, multi-method understanding of future climate impacts on grain production.

Based on these findings, the following agricultural adaptation strategies are recommended: (1) Each cropping area should design precise irrigation regimes based on the estimated optimal SPEI values. (2) As the future impacts of climate change exhibit significant spatial variation, local agricultural departments should formulate differentiated adaptation strategies based on local crop and climate characteristics. For example, in southeastern China where rice is negatively impacted by climate change, a restructuring of cropping patterns should be encouraged. In warming and humidifying regions of Northwest China, rice expansion or increased yield investment could be considered.

## 6. Conclusions

This study provides a refined assessment of climate change impacts on grain yields across East Asia by developing an improved economics–climate model. Our key innovation lies in capturing the non-monotonic relationship between dry and wet conditions and crop production, moving beyond simpler monotonic assumptions. The resultant Yield Impact of Climate Change (YICC) model and the Impact Ratio of Climate Change (IRCC) metric offer a robust quantitative framework for projecting future effects of climate change on grain production under various climate scenarios. The main findings are as follows:

(1) An improved model able to capture the relationship that first increases monotonically and then decreases monotonically between dry and wet conditions and grain production is established, validated, and considered to be well-performed. The model can analyze climate impacts from the perspective of real-word and practical data.
(2) A non-monotonic relationship is observed between wet and dry conditions and grain production in five crop areas. Quantifying the optimal SPEI for major crop areas provides a precise scientific basis for irrigation management, suggesting that practices like alternate wetting and drying can simultaneously enhance water use efficiency and stabilize total yields.
(3) The stark spatial heterogeneity in temperature responses project a coming climatic redistribution of agricultural potential. While warming exacerbates heat stress and shortens growing periods in already warm southern regions, it alleviates thermal constraints and boosts production in colder northern areas. This may precipitate a northward shift in China’s grain production region.
(4) The divergent future impacts highlight varying levels of vulnerability. The negative impact on China’s wheat production, positive impact on maize and the north–south divergence in rice production signal serious may challenge for spatial pattern of traditional food supply in China. In contrast, the minimal impacts projected for Japan and South Korea suggest a more stable production outlook. The estimates provide a quantitative reference for future crop planning.

The implications of these findings are twofold. For policy, our results argue strongly for targeted regional adaptation strategies rather than one-size-fits-all policies. Investment in heat-resistant varieties is paramount in the south, while the north could potentially benefit from a shift towards maize. For research, the YICC model establishes an improved methodology for future impact studies by incorporating essential nonlinear climate responses.

## Figures and Tables

**Table 1:** Cropping area divisions.

Crop	Code	Cropping Area ^1^	Included Districts	Growing Period
Rice	1	Double-cropping rice area in South China (R_SC)	Guangdong, Guangxi, Hainan, Hong Kong, Macao, Taiwan	5–7, 8–10
	2	Single- and double-cropping rice area in Central China (R_CC)	Jiangsu, Fujian, Shanghai, Zhejiang, Anhui, Jiangxi, Hunan, Hubei, Sichuan, Chongqing	5–7, 8–10
	3	Single- and double-cropping rice areas on the Southwest Plateau of China (R_SWPC)	Guizhou, Yunnan, Tibet, Qinghai	5–7, 8–10
	4	Single-cropping rice area in North China (R_NC)	Beijing, Tianjin, Shandong, Hebei, Henan	6–8
	5	Early maturing single-cropping rice area in Northeast China (R_NEC)	Heilongjiang, Jilin, Liaoning	6–8
	6	Single-cropping rice area in dry area of Northwest China (R_DNWC)	Xinjiang, Ningxia, Gansu, Inner Mongolia, Shanxi, Shaanxi	6–8
	7	Japan rice area (R_J)		7–8
	8	Korea rice area (R_K)		7–8
Wheat	1	Winter wheat (autumn sowing) area in northern China (WW_NC)	Shandong, Henan, Hebei, Shanxi, Beijing, Tianjin	(-) 11–5 ^2^
	2	Winter wheat (autumn sowing) area in southern China (WW_SC)	Fujian, Jiangxi, Guangdong, Hainan, Guangxi, Hunan, Hubei, Guizhou, Yunnan, Sichuan, Chongqing, Jiangsu, Anhui, Hong Kong, Macao, Taiwan, Zhejiang, Shanghai	(-) 11–5
	3	Spring wheat (spring sowing) area of China (SW_C)	Heilongjiang, Jilin, Liaoning, Inner Mongolia, Ningxia, Shaanxi, Gansu	5–8
	4	Winter and spring sowing wheat areas of China (WSW_C)	Xinjiang, Tibet, Qinghai	(-) 11–5, 5–8
	5	Japan wheat area (W_J)		(-) 12–5
	6	Korea wheat area (W_K)		(-) 11–5
Maize	1	Spring maize area in northern China (M_NC)	Heilongjiang, Jilin, Liaoning, Inner Mongolia, Shanxi, Shaanxi, Ningxia	5–9
	2	Summer maize area in the Huang-Huai-Hai Plain (M_HHHP)	Hebei, Tianjin, Beijing, Henan, Shandong	7–9
	3	Maize area in the Southwest China Mountains (M_SWCM)	Sichuan, Chongqing, Guizhou, Yunnan	6–8
	4	Maize area in hilly southern China (M_HSC)	Hubei, Anhui, Jiangsu, Shanghai, Zhejiang, Hunan, Jiangxi, Fujian, Guangdong, Guangxi, Hainan, Hong Kong, Macao, Taiwan	6–7
	5	Irrigated maize area in Northwest China (M_NWC)	Xinjiang, Gansu	6–9
	6	Mazie area on the Qinghai–Tibetan Plateau of China (M_QTPC)	Qinghai, Tibet	6–9
	7	Japan maize area (M_J)		5–8
	8	Korea maize area (M_K)		4–8

^1^. The regional boundary is defined by the combined administrative boundaries of all the “included districts”. ^2^. “(-)” represents the month of the previous year.

**Table 2:** Evaluation of extrapolation performance.

Crop Area	TSS1	TSS2	TSS3	Crop Area	TSS1	TSS2	TSS3
	MRE (SDRE)				MRE (SDRE)		
Rice 1	0.9715	0.9857	0.9857	Wheat 4	0.9902	0.9906	0.9906
Rice 2	0.9678	0.9822	0.9822	Wheat 5	0.1061 (0.0874)		
Rice 3	0.9885	0.9793	0.9793	Wheat 6	0.1336 (0.1129)		
Rice 4	0.9945	0.9949	0.9949	Maize 1	0.9402	0.9400	0.9400
Rice 5	0.9917	0.9916	0.9916	Maize 2	0.9908	0.9644	0.9644
Rice 6	0.9406	0.9536	0.9536	Maize 3	0.9616	0.9765	0.9765
Rice 7	0.0464 (0.0398)			Maize 4	0.9736	0.9724	0.9724
Rice 8	0.0401 (0.0340)			Maize 5	0.9211	0.9075	0.9075
Wheat 1	0.9857	0.9810	0.9810	Maize 6	0.9192	0.9678	0.9678
Wheat 2	0.9716	0.9548	0.9548	Maize 7	0.0124 (0.0112)		
Wheat 3	0.9420	0.9635	0.9635	Maize 8	0.0599 (0.0516)		

Note: Numbers in the column of “Crop Area” represent crop code in Table 1. Numbers after “TSS” represent the sequences of experiments.

## Data Availability

All data are freely available from public datasets and websites. Historical daily temperature, downward shortwave radiation flux, SPEI, and PDSI can obtained or calculated from https://psl.noaa.gov/data/gridded/data.cpc.globaltemp.html (accessed on 16 April 2023); https://cds.climate.copernicus.eu/datasets/reanalysis-era5-land-monthly-means?tab=overview (accessed in 1 March 2023); https://spei.csic.es/database.html (accessed on 20 December 2024); and https://crudata.uea.ac.uk/cru/data/drought/ (accessed on 14 September 2024), respectively. Social-economic data of Japan and South Korea can be obtained from https://www.fao.org/faostat/en/#data (accessed on 1 March 2023). Future climate data can be obtained from https://doi.org/10.7917/OFSG3345 (accessed on 1 August 2023) for temperature and radiation and from https://www.ciesin.columbia.edu/data/globaldrought/ (accessed on 31 March 2025) for SPEI.

## Appendix A

### Models Selection

Table A1 shows all models selected to calculate future climate variables under three scenarios.

**Table A1:** Model selection.

Model	Institution
ACCESS-CM2	Commonwealth Scientific and Industrial Research Organisation (CSIRO), ARC Centre of Excellence for Climate System Science (ARCCSS), Australia
BCC-CSM2-MR *	National Climate Center (Beijing), China
CanESM5	Canadian Centre for Climate Modelling and Analysis (CCCma), Canada
CMCC-ESM2	Euro-Mediterranean Center on Climate Change Foundation (CMCC Foundation), Italy
MIROC6/MIROC-ES2L **	Japan Agency for Marine-Earth Science and Technology (JAMSTEC), Atmosphere and Ocean Research Institute, University of Tokyo (AORI), National Institute for Environmental Studies (NIES), RIKEN Center for Computational Science (R-CCS), Japan
MPI-ESM1-2-HR	Max Planck Institute for Meteorology, Germany
MPI-ESM1-2-LR	Max Planck Institute for Meteorology, Germany
MRI-ESM2-0	Meteorological Research Institute (MRI), Japan
NESM3 *	Nanjing University of Information Science and Technology (NUIST), China
NorESM2-LM	CICERO (Center for International Climate and Environmental Research—Oslo), MET Norway (Norwegian Meteorological Institute), NERSC (Nansen Environmental and Remote Sensing Center), NILU (Norwegian Institute for Air Research), UiB (University of Bergen), UiO (University of Oslo), and NORCE (Norwegian Research Centre AS), Norway
NorESM2-MM	CICERO (Center for International Climate and Environmental Research—Oslo), MET Norway (Norwegian Meteorological Institute), NERSC (Nansen Environmental and Remote Sensing Center), NILU (Norwegian Institute for Air Research), UiB (University of Bergen), UiO (University of Oslo), and NORCE (Norwegian Research Centre AS), Norway
TaiESM1	Research Center for Environmental Changes, Academia Sinica, Taiwan, China

Note: * Due to data limitations, this model’s simulation results were not used in the calculation of SPEI. ** Due to data limitations, MIROC6 was used to calculate effective accumulated temperature and shortwave radiation, while MIROC-ES2L was used for SPEI calculation.

## Appendix B

### Data Preprocessing

Since climatic conditions can only directly affect crop growth during the growing period, this study calculated the mean value of each climate variable during the growing period to represent the climate conditions. This study excluded all samples containing missing values. Because the relationship between temperature and crop growth rate is not strictly increasing—excessively high temperatures can inhibit growth—effective accumulated temperature is calculated using a method that simultaneously considers the biological upper limit, lower limit, and optimum temperature, as shown in Equation (A1) [49].

(A1) $$\begin{array}{l} EAT=\sum_{k=1}^{s}e_{k},\\ where:e_{k}=\left\{\begin{array}{l} 0,t_{k}\leq t_{b}\\ t_{k}-t_{b},t_{b}<t_{k}\leq t_{0}\\ \frac{\left(t_{0}-t_{b})(t_{u}-t_{k}\right)}{t_{u}-t_{0}},t_{0}<t_{k}\leq t_{u}\\ 0,t_{k}>t_{u} \end{array}\right. \end{array}$$

where *EAT* represents the effective accumulated temperature (°C·d), *e_k_* is the daily effective temperature (°C); *s* is the total number of days in the growing period; *t_k_* is the daily average temperature on the *k*^th^ day; and *t_b_*, *t*_0_, and *t_u_* represent the lower limit, optimal, and upper limit temperatures (°C) for crop growth, respectively. The lower limit, optimal, and upper limit temperatures for rice, wheat, and corn are shown in Table A2 [50,51].

**Table A2:** Three-point temperatures for rice, wheat and corn.

Crop Type	Lower Limit Temperature	Optimal Temperature	Upper Limit Temperature
Rice	13.5	27.6	35.4
Wheat	0	21.75	37
Maize	6.2	30.8	42.0

Missing data of socio-economic data were estimated according to the methods used in the previous literature [15]. Finally, records from 1991 to 2020 for 34 provincial administrative units in China and all indicators without missing values in Japan and South Korea were selected to form the sample set.

## Appendix C

### Results of Sensitivity Test

Table A3 shows the estimation results of climate variables in the provincial fixed-effect model for China’s crop areas.

**Table A3:** Coefficients of the provincial fixed-effect model.

Crop Area	*γ*_1_	*γ*_2_	*b*	*a*	Crop Area	*γ*_1_	*γ*_2_	*b*	*a*
Rice 1	−0.643 ***	−0.067	−0.044	−0.069 *	Wheat 3	−2.299 *	0.577	0.001	−0.008
Rice 2	−0.224 ***	0.142 **	−0.027 **	−0.040 ***	Wheat 4	−0.834	0.094	0.010	0.062
Rice 3	−0.130	0.702 ***	0.052	0.007	Maize 1	−0.722 **	−0.581 ***	0.049	−0.051 *
Rice 4	0.364	0.147	0.074 ***	−0.013	Maize 2	0.357	−0.348	0.076 ***	−0.067 **
Rice 5	−0.464	−0.042	−0.078 *	−0.012	Maize 3	−0.029	0.694 *	0.031	−0.094 ***
Rice 6	−0.869	0.227	−0.036	0.005	Maize 4	0.222	0.001	0.025	−0.002
Wheat 1	−0.063	0.272 **	0.008	−0.018	Maize 5	0.233	1.208 **	0.072	−0.004
Wheat 2	0.489 **	−0.017	0.073 ***	−0.064 **	Maize 6	−4.679 **	0.145	−0.083	−0.087

Note: “***” indicates *p* < 0.01; “**” indicates *p* < 0.05; and “*” indicates *p* < 0.1 based on the *t*-test.

Table A4 shows the estimation results of climate variables of the model where SPEI is substituted with PDSI for all crop areas.

**Table A4:** Coefficients of the model where PDSI is used to represent C3.

Crop Area	*γ*_1_	*γ*_2_	*b*	*a*	Crop Area	*γ*_1_	*γ*_2_	*b*	*a*
Rice 1	0.121	−0.455	0.016 ***	−0.004 **	Wheat 4	2.088 **	0.863 ***	0.020 *	0.011
Rice 2	−0.483 ***	−0.317 ***	−0.007	−0.003 *	Wheat 5	1.176	0.685 *	−0.027	−0.012
Rice 3	−0.162	0.486 ***	0.012 *	−0.004	Wheat 6	0.126	0.852 **	−0.061 ***	0.010
Rice 4	−0.106	0.740 ***	0.018	−0.006 *	Maize 1	0.107	−1.100 ***	0.032 ***	0.005
Rice 5	0.076	0.002	−0.011	−0.008 ***	Maize 2	−0.099	0.126	0.006	−0.013 ***
Rice 6	1.173 ***	−0.473 ***	0.003	0.005	Maize 3	0.013	−0.282 **	0.005	−0.005 *
Rice 7	−0.074	0.723 ***	−0.023 ***	0.003	Maize 4	0.136	0.295	0.003	0.003
Rice 8	0.230 *	0.108	0.003	−0.001	Maize 5	−0.089	0.975 **	0.015	−0.007
Wheat 1	−0.727 ***	0.287 ***	0.014 **	−0.007 ***	Maize 6	−2.773	0.315	0.011	−0.006
Wheat 2	−0.041	−0.530 ***	0.012 *	−0.001	Maize 7	−0.202	0.190 *	−0.004	−0.002
Wheat 3	−0.387	1.573 **	0.029	−0.001	Maize 8	0.267	−0.513	0.000	−0.004

Note: “***” indicates *p* < 0.01; “**” indicates *p* < 0.05; and “*” indicates *p* < 0.1 based on the *t*-test.
